# Neonatal Dandy–Walker syndrome: a case report

**DOI:** 10.3389/fped.2025.1713294

**Published:** 2026-01-08

**Authors:** Ying Yang

**Affiliations:** Department of Pediatrics, Zigong Fourth People’s Hospital, Sichuan, China

**Keywords:** case report, Dandy–Walker syndrome, early recognition, MRI, neonatal

## Abstract

Dandy–Walker syndrome (DWS) is usually identified prenatally or after hydrocephalus-related symptoms emerge. We report a term male neonate in whom classic DWS was incidentally discovered during admission for purpura and mild tachypnea. Although fetal magnetic resonance imaging (MRI) had suggested vermian agenesis, definitive postnatal brain MRI on day 6 confirmed complete vermian absence, left cerebellar hypoplasia, and a large posterior-fossa cyst communicating with the fourth ventricle. The purpura and respiratory symptoms resolved rapidly with supportive care, and the infant was discharged with full oral feeding at 7 days. This case highlights the need to revisit prenatal imaging when neonates present with seemingly unrelated problems; early postnatal confirmation enables timely family counseling, developmental surveillance, and multidisciplinary follow-up.

## 1 Introduction

Dandy–Walker syndrome (DWS) is a rare neurodevelopmental disorder defined by hypoplasia or aplasia of the cerebellar vermis, cystic dilatation of the fourth ventricle, and an enlarged posterior fossa (1). The global incidence is approximately 1/25,000–1/ 35,000 live births (2). Although its anatomy is well characterized, neonatal presentation remains poorly described: most infants are identified prenatally or later in infancy when hydrocephalus or gross motor delay emerges. A 2017 systematic review of 74 published cases found that over half were incidental discoveries on neuro-imaging performed for unrelated neonatal problems (birth asphyxia, respiratory distress, metabolic acidosis), and purpura or mild tachypnoea had not been reported as the admitting symptom complex (3). We report a term male neonate who was admitted for purpura and mild tachypnea, in whom brain magnetic resonance imaging (MRI) subsequently revealed the classic anatomical features of DWS. This case underscores the importance of revisiting prenatal imaging when common neonatal signs arise and highlights the need for early postnatal confirmation to enable timely family counseling and developmental surveillance.

## 2 Methods

This study is a single-patient retrospective case report. All clinical data, imaging files, and laboratory results were extracted from the electronic medical record of Zigong Fourth People's Hospital on 5 September 2025. No additional tests or interventions beyond routine neonatal intensive care were performed for this report. According to the institutional policy of Zigong Fourth People's Hospital and the Chinese national regulation “Ethical Review of Biomedical Research Involving Human Subjects” (2016), ethical approval is exempted for retrospective, non-interventional case reports. Written informed consent for publication, including clinical data and neuroimaging, was obtained from both parents. A copy of the signed consent form is kept on file and is available for review upon request.

## 3 Clinical case

### 3.1 Prenatal and birth history

The patient was born at 39^+3^ weeks' gestation to a 38-year-old gravida 2 para 2 mother via repeat cesarean section for uterine scarring. The antenatal course was notable for a 32-week fetal ultrasound showing an intracranial cyst and left cerebellar hypoplasia. Fetal MRI confirmed vermis agenesis and abnormal cerebellar architecture. Amniocentesis revealed a normal 46, XY karyotype. Parents elected to continue with the pregnancy after expert consultation. The patient’s Apgar score was 10 at 1, 5, and 10 min, respectively. The patient’s birth weight was 3,430 g (50th percentile), length was 51 cm (50th percentile), and head circumference was 34 cm (50th percentile).

Maternal risk factors included advanced maternal age (38 years), previous cesarean scar, hyperlipidemia, hypercholesterolemia, and first-trimester threatened abortion managed with progesterone. Family history was significant for a prior child who died at 2 years of age due to a serious atrial septal defect.

### 3.2 Presenting illness

At 7 h after birth, a routine ward round revealed generalized purpura that was most prominent on the patient’s face, trunk, and thighs. Mild tachypnea (55 breaths/min) with occasional groans and postfeed regurgitation were noted. There was no fever, cyanosis, or seizures. The newborn was transferred to the neonatal intensive care unit (NICU) immediately.

### 3.3 Initial evaluation

Upon admission, the patient’s vital signs were as follows: temperature, 36.0°C; pulse, 138 beats/min; respiratory rate, 55 breaths/min; blood pressure, 60/42 mmHg; and SPO_2_, 91% on room air. A physical examination revealed diffuse purpura and cold extremities. Capillary refill time was <3 s. The anterior fontanelle was soft and flat. Primitive reflexes were intact.

The laboratory results showed leukocytosis (27.31 × 10^9^/L), neutrophil predominance (83.9%), mild anemia (193 g/L), normal platelet count (184 × 10^9^/L), normal C-reactive protein (CRP; 0.26 mg/L), and markedly increased interleukin-6 (IL-6; 105.37 pg/mL), which confirmed the existence of inflammation. The coagulation profile indicated reduced fibrinogen (1.52 g/L) and prolonged activated partial thromboplastin time (48.7 s), compatible with early-onset consumptive coagulopathy secondary to purpura. Both parameters improved without plasma supplementation. Cardiac enzymes were elevated (creatine kinase (CK) 706 U/L and creatine kinase-MB isoenzyme (CK-MB) 49 U/L), indicating myocardial damage caused by infection, which reverted to normal after 7 days of intravenous fluids. Arterial blood gas revealed compensated metabolic acidosis attributable to respiratory compensation (pH 7.44, base deficit −3.4 mmol/L, and lactate 4.59 mmol/L). The patient’s lactate level reverted to normal after 3 days of intravenous fluids and oxygen.

Immunoglobulin G (IgG) titration indicated transplacental antibody transfer, with increased levels of herpes simplex virus type I IgG antibody (404.01 AU/mL), cytomegalovirus IgG antibody (187.88 AU/mL), and rubella virus IgG antibody (18.92 IU/mL). Blood culture and group B streptococcal PCR were negative.

A lung ultrasound demonstrated bilateral B-lines, which indicated pneumonia. Echocardiography showed mild tricuspid regurgitation but a structurally normal heart.

### 3.4 Hospital course

Empirical intravenous cefotaxime (0.17 g q12h) and supportive care (oxygen via hood with a flow rate of 3 L/min, corrected acidosis with sodium bicarbonate, and intravenous fluids) were initiated. Petechiae began to fade by day 3 and no new lesions appeared. Respiratory effort improved, and oxygen therapy was discontinued on day 3. Enteral feeding advanced from 10 mL q3h to 55 mL q3h without intolerance. Weight nadir was 3.38 kg on day 2 and returned to 3.43 kg by day 7.

**Figure 1:**
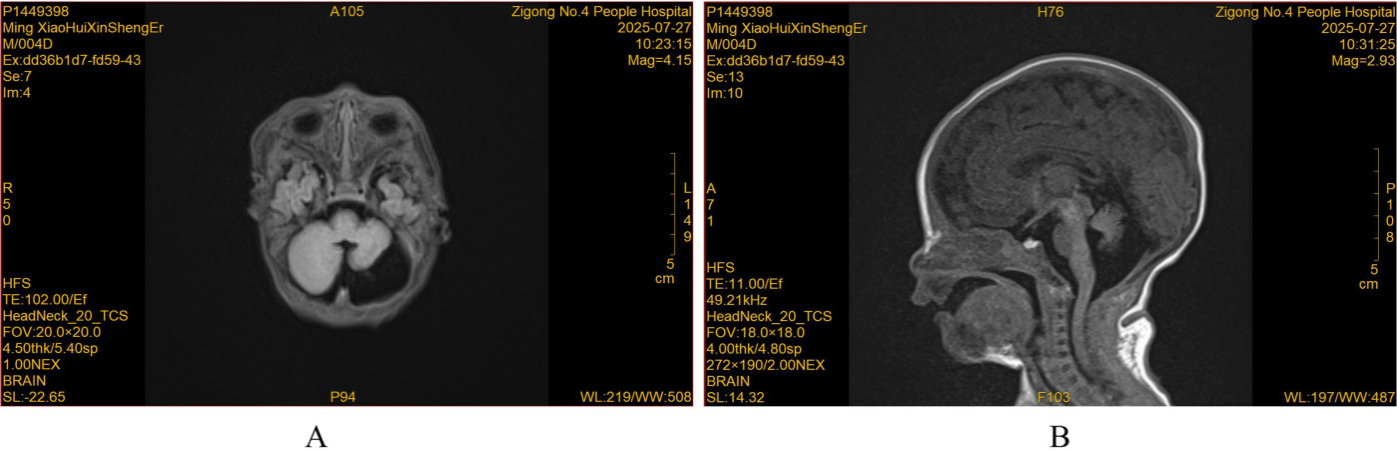
MRI of the brain. **(A)** Coronal scan (T2W1_blade dark fluid): complete absence of the cerebellar vermis and hypoplasia of the left cerebellar hemisphere. **(B)** Sagittal scan (T1W1_qtse dark fluid): posterior fossa cyst communicating with the dilated fourth ventricle.

On day 6, brain MRI was performed because of the prenatal cerebral anomalies. Imaging indicated a complete absence of the cerebellar vermis, marked hypoplasia of the left cerebellar hemisphere, and a posterior fossa cyst communicating with the dilated fourth ventricle (2.8 cm × 2.6 cm × 2.1 cm) (Figure 1). The remaining ventricles, fissures, and sulci were symmetrical, with no abnormalities in size, shape, or signal, and the midline structure was centered. Diffusion-weighted imaging (DWI) showed no definite diffusion-restricted signals in the intracranial area. Video electroencephalography and brain function monitoring resulted in a normal amplitude electroencephalogram with normal background activity, a mature sleep-wake cycle, no burst suppression, and no convulsive seizures. Neurosurgical consultation advised follow-up at a pediatric outpatient clinic.

### 3.5 Outcome and follow-up

After a 7-day hospitalization, the newborn was discharged home with full oral feeding. The discharge diagnoses were neonatal pneumonia, neonatal purpura, hyperlactatemia, abnormal myocardial enzyme spectrum, and DWS. Parents were counseled regarding developmental surveillance, signs of raised intracranial pressure, and early intervention services. Outpatient appointments were scheduled for a neurodevelopmental assessment, lactate recheck, and repeat neuroimaging at 3 months.

We conducted growth and development assessments of the infant 1 month after birth, and the results showed that his overall development level was consistent with his age, and no neurological symptoms or positive signs were found. We will continue to conduct long-term developmental surveillance, which is also important for the child.

## 4 Discussion

This newborn presented with purpura and mild tachypnea, initially raising concerns for sepsis or disseminated intravascular coagulation. Normal blood culture and rapid clinical improvement rendered sepsis unlikely. The petechial rash was attributed to transient coagulopathy of the newborn. However, no significant thrombocytopenia was present. The tachypnea corresponded to neonatal pneumonia and mild metabolic acidosis and was unrelated to the posterior fossa pathology.

The incidental confirmation of DWS underlines the importance of a systematic review of prenatal imaging when neonatal problems arise. The constellation of vermis agenesis, fourth ventricular cyst, and enlarged posterior fossa fulfilled the accepted radiological diagnosis criteria (4). MRI distinguished DWS from an arachnoid cyst (no communication with the ventricle) and mega cisterna magna (intact vermis). Complete vermian absence and upward rotation of cerebellar remnants confirmed the diagnosis (1).

DWS is classified as part of the ciliopathy spectrum, with mutations in genes such as *ZIC1*, *ZIC4*, and *FOXC1* identified in familial cases (5). We offered genetic testing, but the parents in this case declined. Normal parental karyotypes reduce the likelihood of a complex syndrome and most neonates with isolated DWS are asymptomatic. However, associated anomalies (hydrocephalus and cardiac defects) may dominate the early course. Our patient exhibited mild cardiovascular findings (tricuspid regurgitation) that did not require intervention. Elevated lactate and cardiac enzyme levels were interpreted as stress responses to perinatal infection and resolved promptly. Acute neurosurgery was unnecessary and early recognition enabled immediate family counseling and developmental surveillance.

Long-term concerns, including bradykinesia, intellectual disability, and hydrocephalus, have been reported in 30%–50% of survivors (6). Neuroimaging and neurodevelopmental assessment are essential. Surgical cyst fenestration or shunting is reserved for progressive hydrocephalus or a symptomatic posterior fossa cyst (7). Early physiotherapy and developmental monitoring improve functional outcomes (8). Parental education includes recognition of vomiting, lethargy, or macrocephaly as potential indicators of evolving hydrocephalus. Prognosis correlates with the degree of vermis hypoplasia and presence of supratentorial anomalies (9). In this clinical case, normal supratentorial structures and electroencephalogram background may predict a more favorable outcome. Regular follow-up will clarify the neurodevelopmental trajectory. Furthermore, this case underscores the need to consider major congenital malformations even when neonatal symptoms appear disconnected from the central nervous system.

## 5 Conclusions

This case demonstrates that classic DWS can be discovered incidentally when neonates present with apparently unrelated problems such as purpura or mild tachypnea. Systematic re-evaluation of prenatal imaging, prompt postnatal MRI, and early multidisciplinary follow-up enabled accurate diagnosis, parental counseling, and developmental surveillance before symptoms of hydrocephalus or motor delay emerged. More broadly, the findings underscore that major congenital malformations should remain part of the differential diagnosis even when neonatal symptoms appear unrelated to the central nervous system.

## Data Availability

The original contributions presented in the study are included in the article/Supplementary Material, further inquiries can be directed to the corresponding author.
